# CLCN5 inhibits tumorigenesis and fatty acid accumulation in clear cell renal cell carcinoma by regulating Enoyl CoA hydratase and 3-Hydroxyacyl CoA dehydrogenase

**DOI:** 10.7150/ijms.105969

**Published:** 2025-07-19

**Authors:** Tiexi Yu, Weiquan Li, Xiangui Meng, Wei Yang, Hailong Ruan, Wen Xiao, Xiaoping Zhang

**Affiliations:** 1 Department of Urology, Union Hospital, Tongji Medical College, Huazhong University of Science and Technology, Wuhan, 430022, China.; 2 Shenzhen Huazhong University of Science and Technology Research Institute, Shenzhen 518000, China.; 3 Institute of Urology, Tongji Medical College, Huazhong University of Science and Technology, Wuhan 430022, China.

**Keywords:** clear cell renal cell carcinoma(ccRCC), fatty acid metabolism, tumor metabolism

## Abstract

Clear cell renal cell carcinoma (ccRCC), a globally prevalent and highly aggressive malignancy, is characterized by abnormal lipid accumulation and high morbidity. However, the complex pathological mechanisms underlying its development remain largely unexplored, necessitating further research efforts. In this study, we employed Weighted Gene Co-expression Network Analysis (WGCNA) and identified Chloride Voltage-Gated Channel 5 (CLCN5), a member of the CIC family, as a potential hub gene involved in fatty acid degradation. Our findings suggest that downregulated CLCN5 was negatively correlated with the malignant characteristics and prognosis of ccRCC. *In vitro* experiments demonstrated that CLCN5 overexpression significantly impacts fatty acid oxidation and inhibits tumor proliferation, metastasis, migration, and invasion in ccRCC. Mechanistically, CLCN5 restrains the proliferation and migration of ccRCC cells by decreasing lipid accumulation through the effects of Enoyl CoA hydratase and 3-Hydroxyacyl CoA dehydrogenase (EHHADH). Collectively, these findings suggest that CLCN5/EHHADH-mediated fatty acid metabolism could be a potential strategy for ccRCC treatment.

## Introduction

Renal cell carcinoma (RCC) is a highly aggressive tumor affecting the urinary system, with a rising global incidence of 2-3% among all malignant cancers 1. In 2024, approximately 81610 Americans were diagnosed with RCC, leading to an estimated 14390 deaths 2. RCC encompasses various histologic and genetic subtypes 3, 4, with clear cell RCC (ccRCC) representing 75% of cases 5, 6. The primary pathological feature of renal clear cell carcinoma is the accumulation of significant lipid droplets. Recent studies have underscored the critical role of abnormal lipid accumulation in the initiation and progression of ccRCC^7^. Elucidating the mechanisms underlying lipid buildup may offer deeper insights into the pathogenesis of renal cancer.

The Chloride Voltage-Gated Channel 5 (CLCN5) gene, located at Xp11.22, encodes a protein that is part of the chloride ion channels and transporters family, known as the CIC family. This protein is primarily localized to endosomal membranes and is believed to facilitate the uptake of albumin by the renal proximal tubule. Mutations identified within this gene have been associated with the development of Dent disease and renal tubular disorders, often accompanied by nephrolithiasis. The upregulation of CLCN5, induced by interleukin-4 in leukemia, has resulted in the activation of various oncogenes 8. CLCN5 has also been implicated in proteinuria and chronic lymphocytic leukemia. However, its role in RCC remains inadequately explored. Further research is required to elucidate the potential involvement of CLCN5 in the tumorigenesis of RCC.

ccRCC was considered a metabolic disease as its intracellular lipid droplets (LDs) mainly composed of triglycerides and cholesterol esters 9, 10. Previous studies had shown the accumulation of lipid resulted from a block of degradation in ccRCC^11^, ^12^. Preliminary investigations conducted by the researcher's team have substantiated that enhancing lipid catabolism plays a pivotal role in markedly suppressing the proliferation, migratory potential, and invasive capabilities of clear cell renal cell carcinoma (ccRCC) 13-15. Enoyl-CoA hydratase and 3-hydroxyacyl CoA dehydrogenase (EHHADH), related to fatty acid degradation 16, 17, was a key gene in fatty acid metabolism of hepatocellular carcinoma 18. Currently, it remains unclear whether EHHADH participates in lipid degradation within ccRCC and influences its malignant progression.

This article provides a systematic exploration of CLCN5 in ccRCC. Our findings indicate that downregulated CLCN5 serves as a potential biomarker for diagnosing and predicting the prognosis of ccRCC. *In vitro* experiments demonstrate that modulating CLCN5 expression could significantly impacts fatty acid oxidation, tumor proliferation, metastasis, migration, and invasion in ccRCC. Mechanistically, CLCN5 inhibits fatty acid accumulation by upregulating EHHADH expression. These insights enhance our understanding of the involvement of CLCN5/EHHADH-mediated fatty acid metabolism in ccRCC.

## Materials and Methods

### Bioinformatics analyses

Characterized by the median level of CLCN5 expression, the ccRCC tissues were segregated into two distinct groups; Then, we used gene set enrichment analysis (GSEA) to identify enriched pathways 19. p < 0.05 and false discovery rate (FDR) < 0.25 were considered statistically significant. Additionally, the differential expression of genes between the two groups was analyzed using the Kyoto Encyclopedia of Genes and Genomes (KEGG) and Gene Ontology (GO) methods in R 4.1.3. We employed various bioinformatics tools and networks to analyze the data and generate visual representations, such as GEPIA, LinkedOmics, and Venny 20, 21.

### Dataset acquired and WGCNA

TCGA-KIRC (Version: 2017-10-13) data were downloaded from UCSC Xena browser as other cancers 22, 23. GEO database (GSE44035, GSE66272, GSE53757), which included gene RNA sequencing and mutations, and CNA data, were downloaded and analyzed by R 24. The “limma” package was utilized to confirm differentially expressed genes (DEGs), and we chose |logFoldChange| ≥1.5 as the criteria for DEGs 25. Weighted Gene Co-expression Network Analysis (WGCNA) was a robust computational approach that effectively detects cohesive gene modules exhibiting high correlations. WGCNA discovered the critical regulators of governing biological processes and the elucidation of novel genetic associations in intricate diseases 26, 27. All of the above were performed by R 4.1.3^28^.

### Survival analysis

According to the median level of CLCN5 mRNA, ccRCC tissues was divided into two arrays. Kaplan-Meier plots and receiver operating characteristic (ROC) curves analyzed the differences in overall survival (OS) and disease-free survival (DFS) between the two groups 29. The results were believed to be statistically significant if the p-value < 0.05. The software used for generating the plots was GraphPad 8.01.

### Cell culture

High glucose Dulbecco's Modified Eagle's Medium (Gibco, USA) with the addition of 10% fetal bovine serum (Gibco, USA) was used to culture cells. Two human normal kidney cells (HK2, 293) and four tumor cells (786O, Caki-1, OSRC-2, A498) were maintained at 37.3°C with 5% CO2 30.

### ccRCC tissue samples

This study recruited a cohort of 33 ccRCC patients from Urology, Wuhan Union Hospital, China. This study complied with the regulations the Human Research Ethics Committee of Huazhong University of Science and Technology set forth. It adhered to the principles outlined in the Declaration of Helsinki. RNA was isolated from 24 pairs of samples and quantified using quantitative real-time polymerase chain reaction (qRT-PCR). In comparison, proteins were acquired from 8 pairs of samples and analyzed by western blot 31. Additionally, a couple of tissues underwent immunohistochemical (IHC) analysis. 33 Clinical information on the pairs of samples was attached in the table that follows.

### RNA extraction and RNA reverse transcription

Tissue and cell RNA extraction were carried out as previously described 32, 33. The cell precipitate was collected, washed with PBS, and transferred to enzyme-free EP tubes. And then, 1ml of TRIzol was added. The subsequent steps were consistent. RNA reverse transcription: The composition of the 20 µl reaction system is 4 µl HiScript II Q Select RT SuperMix (Abclonal, China), 12.0 µl RNase-free H_2_O, and 4.0 µl. The obtained RNA is according to the above steps.

### qRT‑PCR

After reverse transcribed into cDNA, the cDNA was utilized for qRT-PCR analysis using a fluorescent quantitative PCR instrument (Analytik Jena AG, Germany) and SYBRbGreen mix (Abclonal, China). GAPDH was utilized to be internal control. The qRT-PCR assays were carried out in triplicate or quadruplicate. This study's forward, or reverse primers were acquired from Sangon Biotech (Shanghai). The specific primer sequences utilized for qPCR are presented in the study.

CLCN5:

Forward: 5′-ATAGGCACCGAGAGATTACCAA-3′

Reverse: 5′-CTAACGAACCTGATAAAAGCCCA-3′

GAPDH:

Forward: 5′-ATAGGCACCGAGAGATTACCAA-3′

Reverse: 5′-CTAACGAACCTGATAAAAGCCCA-3′

EHHADH:

Forward: 5′-AAACTCAGACCCGGTTGAAGA-3′

Reverse: 5′-TTGCAGAGTCTACGGGATTCT-3′

PDK2:

Forward: 5′-GAGGACCACCGGACTCTAAG-3′

Reverse: 5′-GTGGGCACCACGTCATTGT-3′

ACBD5

Forward: 5′-GCACGAGACTAGGTTTGAGGC-3′

Reverse: 5′-GTGAACTCCAAGCATCCCATTTA-3′

PGC1A:

Forward: 5′-AAACTCAGACCCGGTTGAAGA-3′

Reverse: 5′- TTGCAGAGTCTACGGGATTCT-3′

PARA:

Forward: 5′-GAGGACCACCGGACTCTAAG-3′

Reverse: 5′-GTGGGCACCACGTCATTGT-3′

UCP1:

Forward: 5′-GCACGAGACTAGGTTTGAGGC-3′

Reverse: 5′-GTGAACTCCAAGCATCCCATTTA-3′

PTH:

Forward: 5′-AAACTCAGACCCGGTTGAAGA-3′

Reverse: 5′-TTGCAGAGTCTACGGGATTCT-3′

CPT1A:

Forward: 5′-GAGGACCACCGGACTCTAAG-3′

Reverse: 5′-GTGGGCACCACGTCATTGT-3′

SLC27A2:

Forward: 5′-TTTCCGCCATCTACACAGTCC-3′

Reverse: 5′-CGTAGGTGAGAGTCTCGTCG-3′

### Protein extraction, western blot

Antibodies utilized in the study were as follows: anti-CLCN5 (Abclonal, A5707, 1:1000), anti-GAPDH (Proteintech, 20536-1-AP, 1:1000), anti-ACBD5 (Abclonal, A10595, 1:1500), anti-EHHADH (Abclonal, A5517, 1:1000). The collected ccRCC samples and cells were processed for protein extraction using a combination of RIPA lysis buffer (Beyotime, China), PMSF, and protease inhibitor cocktail. The protein extraction and western blot were carried out as previously described 32, 33.

### IHC assay

The IHC assays were conducted following the previously established protocol. Briefly, tissue samples were fixed using 4% paraformaldehyde and underwent a series of steps, including dehydration, paraffin embedding, sectioning, deparaffinization, and rehydration for antigen retrieval. The tissue sections were then blocked with bovine serum albumin and incubated with primary antibodies, followed by secondary antibodies.

### Transient transfection

The expression of CLCN5 was modulated using plasmids overexpressing the gene, siRNA or shRNA (GV298 vector containing small hairpin RNA, shRNA). Genechem (Shanghai, China) and Genepharma (Soochow, China) constructed and synthesized the plasmids and siRNA.

Cells were transfected at a density growth between 50 and 60 percent. The transfection procedure was performed by introducing three µg of the overexpression plasmids or siRNA into individual wells of a 6-well cell culture plate. This was achieved by separately combining three µg of plasmids with 50 µl of Opti-MEM in one tube and mixing 3 µl of Lipofectamine 3000 reagent (Invitrogen, CA, USA) with 50 µl of Opti-MEM (Invitrogen, CA, USA) in another tube. Following a 30-minute incubation period to allow for complex formation, the contents of the two small tubes were combined and added to each well of the 6-well cell culture plate. Fluid changes at 8-12 hours, depending on cell growth. The cells were subjected to a 48-hour transient transfection with overexpression plasmids (CLCN5 or vector), siRNAs, or shRNAs.

### Cell proliferation assays

Cell proliferation assays were performed after executing a transient transfection lasting at least 48 hours. The cellular culture was maintained in a 96-well plate at 1000 cells per well density. The proliferation analysis employed the CCK-8 kit acquired from YEASEN, China, with a 1:10 dilution in serum-free medium stipulated by the manufacturer's guidelines. The assays were conducted at 24-hour hours, spanning four days. The obtained data were represented in optical density (OD450) readings across the four days and were subsequently visualized using GraphPad Prism software.

### Cell migration and invasion assays

The migration and invasion capacities of cells were evaluated through the transwell assays. The experiments were conducted at least three times independently with serum-starved cells (A498: 20000; 786O 20000) used for the migration assays and double the number of cells used for the invasion assays. For the migration assay, 20,000 cells were added to the top chamber of a 24-well transwell (Corning, USA) and cultured in a serum-free medium for 24 hours. 600 µL of medium with 10% FBS was added to the bottom chamber. After incubation, the cells were fixed in 20% methanol for 25 minutes, stained with 0.5% crystal violet solution for 30 minutes, and then rinsed thrice. After removing any cells on the upper surface, the number of cells in the chamber's lower surface was numbered and imaged under a 200 × light microscope (Olympus). A similar protocol was followed for the invasion assay, except cells were required to invade the Matrigel matrix (Thermo) and pass through the transwell membrane. The assays were conducted following the previously described protocol.^32^

### Colony formation assays

In 6-well plates, A total of 1000 cells each of A498 (Vector, CLCN5, si-NC, si2-CLCN5, si1-CLCN5) and 786-O (Vector, CLCN5, si-NC, si2-CLCN5, si1-CLCN5) were cultured. The cells were cultured for approximately seven days, then fixed for 30 minutes. PBS was used to wash wells and then stained with crystal violet for 40 minutes. Then, taking pictures for analysis and statistics.

### Wound healing assay

The cell cultures were maintained in 6-well plates and were monitored for cell confluence until 100% fusion was achieved. Subsequently, the cells were subjected to a standardized wound generation procedure, creating equivalent wound sizes for each sample. The resulting wounds were visualized and captured 24 hours post-wounding to monitor the healing process.

### Triglyceride determination

To determine the triglyceride content within the cancer cells, a triglyceride determination kit (Nanjing Jiancheng Bioengineering Institute, China) was employed as per the manufacturer's instructions. Cells were initially seeded in a 10 cm dish at 60% confluency. After incubating for 24 hours, the cell pellet was collected from the 10 cm dish. Subsequently, 0.9 mL of Triton X-100 (#P0096, Beyotime) was added to the cell pellet from the 10 cm dish, and mechanical homogenization was performed. The homogenate was centrifuged at 600 ×g for 5 minutes, yielding a supernatant. This supernatant was utilized to assess the triglyceride (TG) content by the instructions provided by the Triglyceride assay kit.

### Oil red O staining

The proliferation and viability of cells were studied by seeding 1000 cells of both A498 and 786-O lines into separate wells of a 6-well plate and allowing them to reach approximately 30% confluence. Then 10% formaldehyde was subjected to fixation for a minimum of 20 minutes, washed, and stained with Oil Red O (Biotechnology) for 30 minutes.

### Fluorescence microscopy analysis

Cells were cultured in 96-well plates. When cell confluence reached approximately 30%, they were treated with a BODIPY (MCE, HY-W090090) working solution (10 µM BODIPY in PBS) for 30 minutes at 37°C. Following this, the cells were stained with DAPI (D9542, Sigma, USA). Fluorescence images were then captured using a fluorescence microscope at 200x magnification.

### Flow cytometry analysis

Cells were cultured in 12-well plates. Once cell confluence reached approximately 80%, the cells were treated with a BODIPY working solution (10 µM in PBS) for 30 minutes at 37°C. Following treatment, the cells were trypsinized, centrifuged, and resuspended in 200 µL PBS per well. Neutral lipid levels were assessed by flow cytometry using the FITC channel.

### Statistical analyses

The results were displayed as mean and Standard Error of Mean (SEM). Statistical comparisons between groups were performed by Student's t-test or paired Student's t-test. Pearson's chi-squared test analyzed the relationship between CLCN5 expression and tumor grade and stage in ccRCC samples. Univariate and multivariate Cox regression analyses were conducted to assess the association between tumor grade and stage and OS, with CLCN5 mRNA expression levels as a binary variable and OS as the dependent variable. SPSS 25.0 was utilized to perform the statistical analysis. p < 0.05 was considered statistically significant. This study's experiments were repeated thrice, and the data were analyzed using GraphPad Prism software. (California, USA), as previously reported 34. The qRT-PCR data of ccRCC samples were presented as mean values, while the rest of the data was represented as mean ± SEM.

## Results

### WGCNA identified CLCN5 as a hub gene in TCGA-KIRC

**Figure 1:**
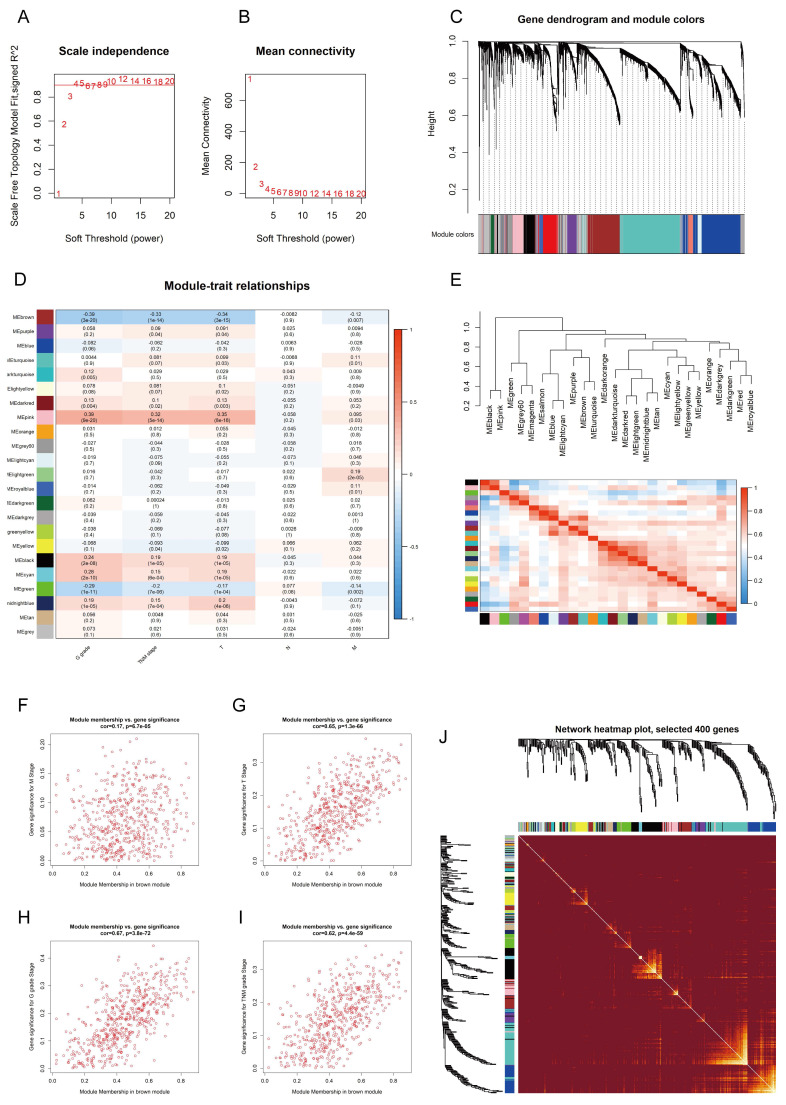
The hub genes of ccRCC were identified using WGCNA. A, B: Scale independence and mean connectivity were analyzed for various soft-thresholding power C: The dendrogram and identified module colors of DEGs were based on TOM. D: The relationships between 25 modules and clinicopathological features. E: The correlation heatmap between each identified module. F-I: The brown module was analyzed for correlation with TNM stage, T stage, Stage, M stage, and G grade.

To identify the hub genes associated with ccRCC from the TCGA-KIRC dataset, firstly, we employed the "limma" package to filter 6,435 DEGs from the gene set using a defined cutoff criterion (|log_2_FoldChange| ≥ 1.5). Then, we applied WGCNA to confirm the modules closely associated with clinical traits. 25 modules were revealed, and the scale-free network was established with a soft threshold β = 4 (Fig. 1A, B). We identified and integrated 25 modules based on DEGs (correlation coefficient < 0.8) (Fig. 1C, E). Following an extensive analysis, it was determined that the brown module exhibited a robust negative correlation with T-stage, N-stage, and G-stage of kidney cancer (Fig. 1D, Fig. 1F-I, Fig. S1A). A correlation heatmap was generated to reflect the relationship between the top 400 selected genes in all modules (Fig. 1J). In addition, GO and KEGG pathway analysis was performed to explore the biological functions and pathways related to the brown module. The brown module was associated with fatty acid degradation (Fig. S1J, K). Subsequently, the WGCNA algorithm was employed to ascertain the hub gene within this module, leading to the identification of CLCN5 as the core gene.

**Figure 2:**
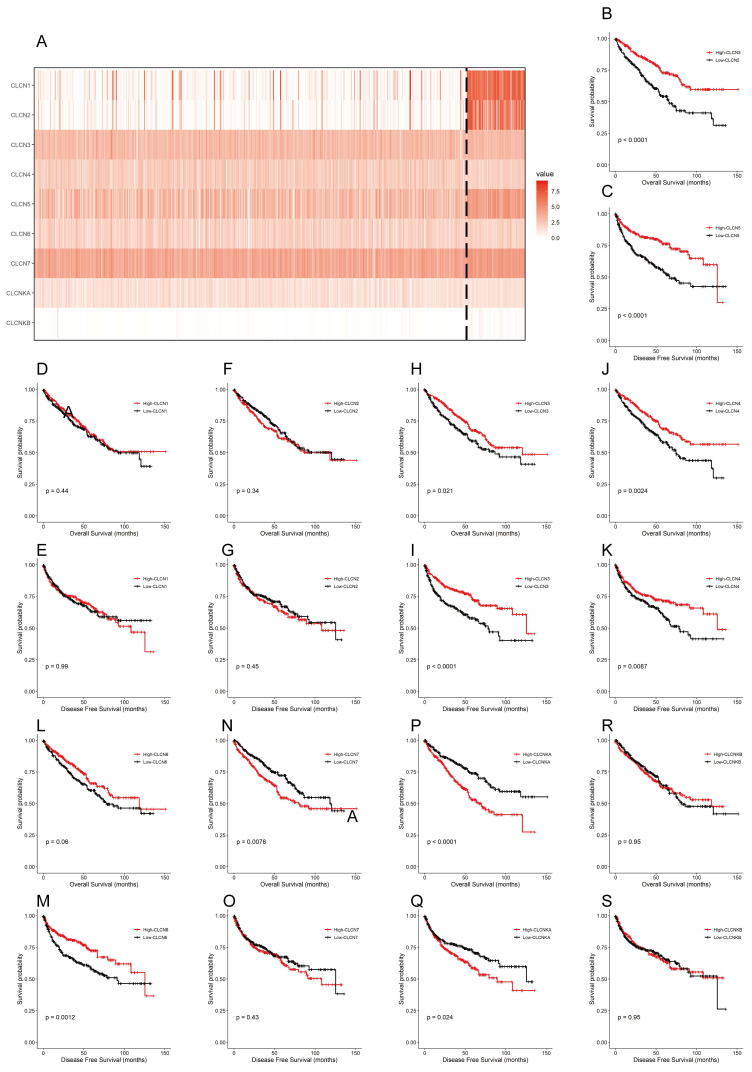
The expression and OS, DFS of nine genes of the CIC family. A: A heatmap of the expression levels of nine CIC family members (CLCN1, CLCN2, CLCN3, CLCN4, CLCN5, CLCN6, CLCN7, CLCNKA, CLCNKB) in TCGA ccRCC. B-S: the Kaplan-Meier of the nine CIC family genes. OS refers to the probability of surviving for a specific period, whereas DFS refers to the likelihood of not having a recurrence or disease progression during a particular period.

Currently, the differential expression and roles of CLCN5 and other members of the CLCN family in ccRCC have not been extensively elucidated. The findings revealed that within the TCGA-KIRC dataset, only 5 genes (CLCN1, CLCN2, CLCN5, and CLCNKB) exhibited differential expression with reduced levels, whereas the other 4 genes (CLCN3, CLCN4, CLCN7, and CLCNKA) displayed no significant differences (Fig. 2A). Furthermore, Kaplan-Meier analyses for OS and DFS demonstrated that CLCN1, CLCN2, and CLCNKB expression levels in ccRCC were not prognostic indicators (Figures 2B-2S). Consequently, our focus shifted to CLCN5 as a promising biomarker candidate for ccRCC, prompting additional investigation.

### A significant relationship was found between CLCN5 expression level and clinicopathological characteristics

**Figure 3:**
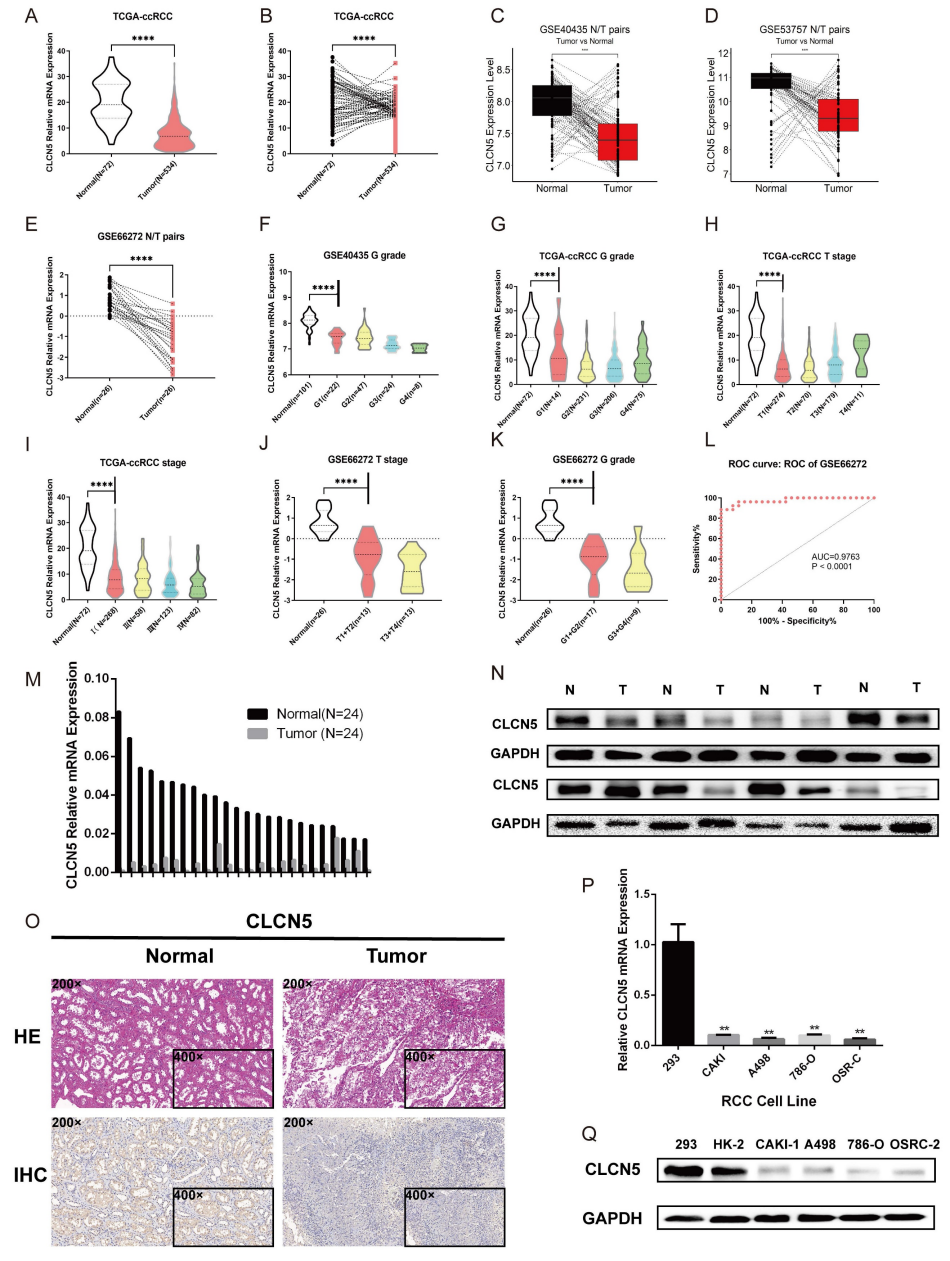
The relationship between CLCN5 and clinical traits, as well as its overexpression. A: Publicly available datasets indicate that CLCN5 is expressed at lower tumor levels than normal tissues. B-D and F-K: Compared to cancer-adjacent tissues, CLCN5 expression is low in cancer tissues, and its expression is positively related to various clinicopathological factors. L The receiver operating characteristic curve of CLCN5. M-Q: CLCN5 is low-expressed in ccRCC tissues and cell lines. qRT-PCR and immunoblotting tests were used for these analyses, as well as HE and immunohistochemical analyses. Error bars present mean ± SEM.

The expression profiles of CLCN5 in multiple GEO datasets of kidney cancer (GSE44035, GSE66272, and GSE53757) were further investigated. We initially assessed the CNAs of CLCN5, and no genetic alterations were present in the TCGA-KIRC (Fig. S1I). Tumor tissue transcriptome analysis revealed reduced CLCN5 expression in both the TCGA-KIRC and GEO datasets (Fig. 3A-E). Correlation analysis reveals a negative correlation between CLCN5 and adverse clinical staging (Fig. 3F-I), consistent with findings in other GEO datasets (Fig. S1. B, C, Fig. 3J, K). Furthermore, diminished expression of CLCN5 was associated with a reduced overall survival (OS) and higher tumor clinicopathological grade (Table 1). Univariate and multivariate analyses indicate that CLCN5 is a crucial independent prognostic indicator for ccRCC (Table 2). Moreover, the ROC curve analysis demonstrated that CLCN5 exhibits significant diagnostic potential in tumor tissues (Fig. 3L and Figure S1H), as well as subgroups of tumor stages and grade subgroups (Figure S1D-G).

### CLCN5 expression was downregulated in samples from ccRCC and renal cancer cells

To validate the aberrant expression of CLCN5, we conducted expression analyses in both clinical specimens and RCC cell lines. The qRT-PCR and WB analyses revealed that the expression of CLCN5 was significantly decreased in tumor samples compared to controls (Fig. 3, M, N). Additionally, the results from the IHC analysis of tumor and tumor-adjacent tissue pairs showed the downregulation of CLCN5 in cancer tissues (Fig. 3O). Moreover, CLCN5 expression was notably downregulated in renal cancer cell lines relative to the normal renal cell line 293 (Fig. 3P, Q). These findings suggest that reduced CLCN5 expression in ccRCC may function as a tumor suppressor.

### Restored CLCN5 expression inhibits RCC cell growth, migration, and invasion

**Figure 4:**
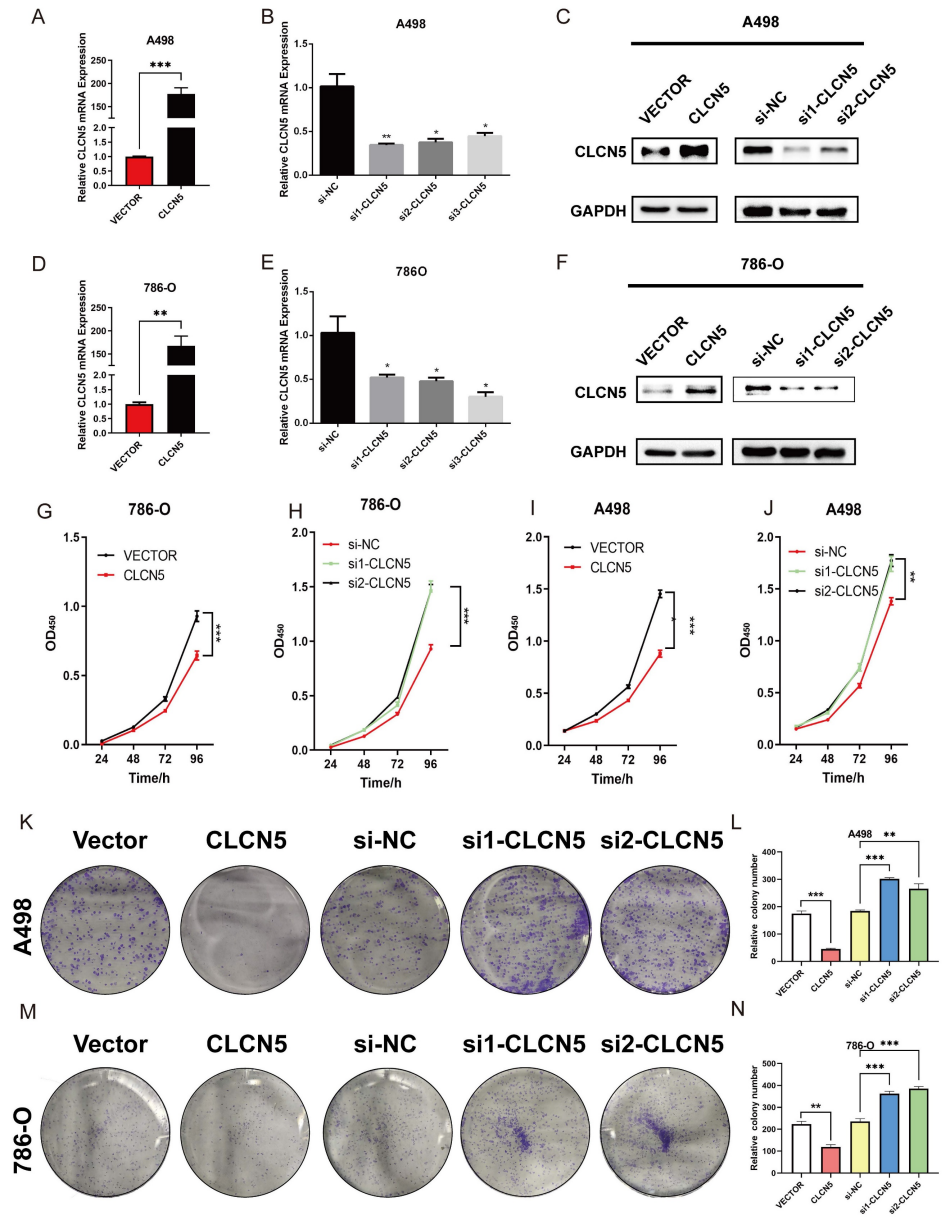
CLCN5 facilitated the tumor proliferation *in vitro*. A, B, D, E The mRNA levels of CLCN5 in tumor cells with CLCN5 knockdown or overexpression were verified by qRT-PCR. C, F: The expression of CLCN5 was confirmed by WB. G-J: The cell proliferation assay of tumor cells was conducted for four days after depleting or overexpressing CLCN5. K-N The capacity of colony formation in tumor cells with CLCN5 overexpression or knockdown was assessed by enumerating the number of colonies generated after seeding 1000 cells in a culture dish for 7 days. The experiments were repeated thrice.

**Figure 5:**
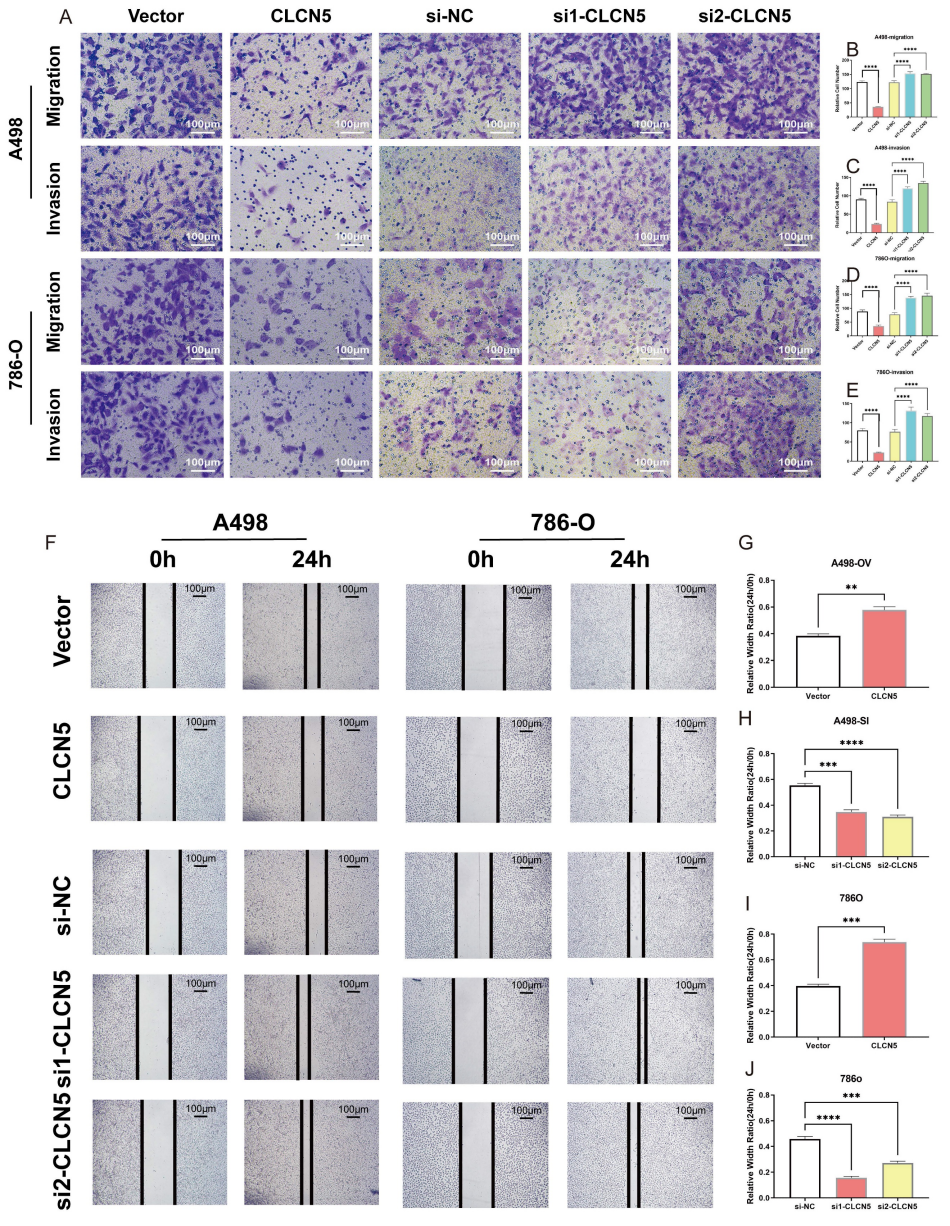
The impact of CLCN5 in promoting migration and invasion of ccRCC *in vitro*. A-E: The transwell assays were conducted to evaluate the cell migration and invasion ability of tumor cells with CLCN5 overexpression or knockdown (Magnification: 200×, n=3 per group). F-J: The migration ability was assessed using wound healing assays in both CLCN5 overexpression and knockdown tumor cells. Images were captured after 24 hours.

To elucidate the potential role and biological effects of CLCN5 in renal cell carcinoma, we conducted *in vitro* functional experiments. Considering the diminished expression of CLCN5 in renal cancer tissues and cell lines (A498 and 786-O), we initially elevated its expression in these cells through plasmid transfection. Additionally, we further reduced CLCN5 levels by introducing siRNAs (Fig. 4A, B, D, E). The cell proliferation assay demonstrated that restored CLCN5 expression in A498 and 786-O cells hindered their proliferation, whereas its downregulation induced the opposite effect (Fig. 4G-4J). Colony formation assays corroborated these findings (Fig. 4K-4N). Additionally, migration and invasion assays revealed a negative correlation between CLCN5 levels and both cell migration and invasive capabilities (Fig. 5A-5E). Wound healing assays further substantiated this conclusion (Fig. 5F-5J). Collectively, these results indicate that restored CLCN5 significantly suppresses the proliferation, migration, and invasion of ccRCC.

### CLCN5 alleviated lipid accumulation in ccRCC

**Figure 6:**
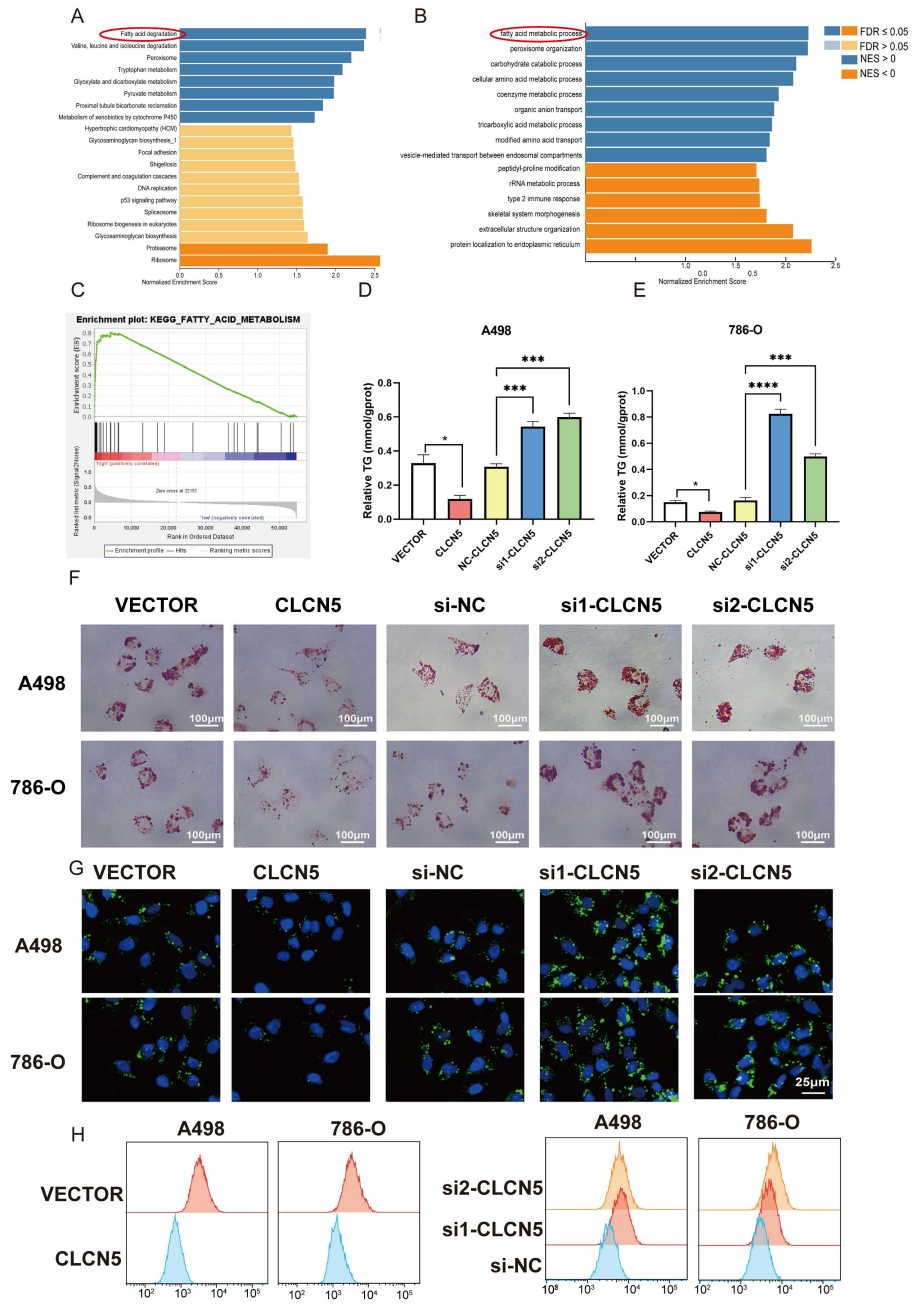
CLCN5 inhibited fatty acid oxidation and accelerated lipid accumulation. A and B: GO and KEGG analysis of CLCN5. C: Gene Set Enrichment Analysis of CLCN5 through the TCGA-KIRC. D and E: Oil Red O staining was utilized to assess lipid accumulation in knockdown and overexpressed CLCN5 tumor cell and their control. F: The amounts of triglycerides were detected using a triglyceride assay kit. G: Fluorescence microscopy analysis with BODIPY. H: Neutral lipid levels were also assessed by flow cytometry.

The hallmark histopathological characteristic of renal clear cell carcinoma is the prominent accumulation of lipid droplets within tumor cells. Thus, we aim to explore the potential role of CLCN5 in modulating lipid metabolism—specifically, either the synthesis or degradation pathways—in ccRCC through functional enrichment analysis. Remarkably, both GO, KEGG analyses (depicted in Fig. 6A, 6B, and S3A-E) and GSEA analysis (Fig. 6C) have demonstrated a markedly correlation between CLCN5 and the metabolic pathways of fatty acid metabolism as well as lipid degradation. Here, we observed a significant reduction in triglyceride levels in ccRCC cell lines following the overexpression of CLCN5, as evidenced by triglyceride assays (Fig. 6D and 6E). Furthermore, diminished intracellular lipid accumulation was corroborated by oil red staining (Fig. 6F) and fluorescence microscopy analysis with BODIPY (Fig. 6G). Neutral lipid levels were also assessed by flow cytometry (Fig. 6H). Next, we constructed stable knockdown CLCN5 renal cancer cell lines (Fig. S4A) and showed the same results with siRNA in fluorescence microscopy analysis flow cytometry (Fig. S4B and 4C).

Collectively, these findings indicate that downregulation of CLCN5 promotes lipid accumulation in ccRCC, whereas reinstating CLCN5 expression may impede tumor progression through enhanced lipid degradation.

### CLCN5 facilitated fatty acid oxidation and diminished the lipid accumulation via EHHADH

**Figure 7:**
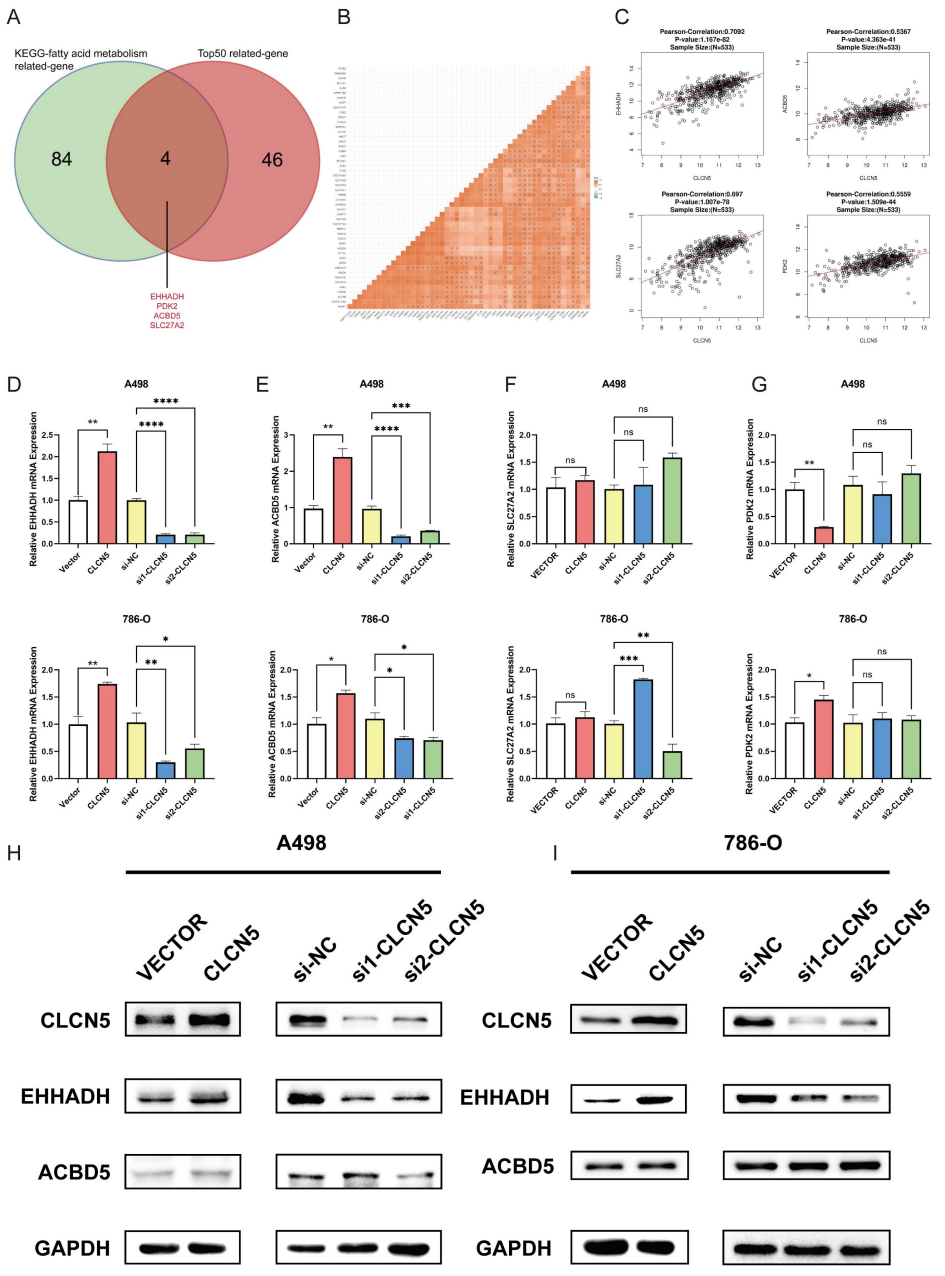
A: The veen diagram of the top 50 genes related to the CLCN5 and KEGG-fat acid metabolism dataset. B and C: The correlation heatmap of the top 50 genes related to CLCN5. G: The correlation between EHHADH, SLC27A2, PDK2, ACBD5, and CLCN5. D and G mRNA levels of four genes (EHHADH, ACBD5, SLC72A2, PDK2) in tumor cells with CLCN5 upregulation and downregulation. H and I: The western blot results of EHHADH and ACBD5 in up-regulated and down-regulated CLCN5 in A498 and 786-O cell lines.

To delve into the precise mechanism by which CLCN5 modulates lipid metabolism, we initially examined established genes implicated in fatty acid metabolism: UCP1, CPT1A, PPARA, PTH, and PGC1A, all of which have been previously documented reported 35-37. However, our results revealed that the expression levels of these genes remained unaltered in both CLCN5-overexpressing and CLCN5-knockdown ccRCC cells relative to negative controls (Fig. S2). Subsequently, we extracted all genes from the KEGG Fatty Acid Metabolism dataset and identified the top 50 CLCN5-associated genes using GEPIA. A correlation heatmap illustrating these 50 genes' relationship with CLCN5 is provided (Fig. S3F). Notably, four genes (EHHADH, PDK2, SLC27A2, and ACBD5) were consistently present in both datasets (Fig. 7A). Further analysis revealed a positive correlation between CLCN5 and these four genes within both the TCGA-KIRC dataset and GEPIA (Figs. 7B and 7C). We then test the expression levels of EHHADH, PDK2, and SLC27A2 in CLCN5-overexpressing and knockdown ccRCC cells. We observed significant alterations in the mRNA levels of EHHADH and PDK2, whereas negligible changes were noted in SLC27A2 expression in the A498 cell line. Additionally, PDK2 expression remained unchanged in CLCN5-knockdown cells but varied in cells with CLCN5 overexpression (Figs. 7D-7G). These findings led us to hypothesize that EHHADH and ACBD5 may be influenced by CLCN5 regulation. However, it was demonstrated that the overexpression or knockdown of CLCN5 in A498 and 786O cells altered EHHADH protein levels, whereas ACBD5 expression remained unaffected (Fig. 7H and 7I). Consequently, we posit that CLCN5 suppresses fatty acid accumulation by upregulating EHHADH, consistent with its established regulatory function in lipid metabolism.

## Discussion

The advancement of research on the pathophysiology of advanced ccRCC had enriched the clinical treatment and anti-tumor reagents 38, 39. The 5-year survival rate of ccRCC patients remained at only 10.5% according to a multicenter experience 40. Prognostic models that integrate multiple effective biomarkers had shown promising results in providing more accurate and personalized predictions of patient survival outcomes 41, 42. Therefore, new biomarkers discovering holds great promise for ccRCC patients.

In our study, we provided multiple evidences for the importance of CLCN5 in the pathogenesis of ccRCC. WGCNA analysis revealed a significant negative correlation between the brown module and the T-stage, N-stage, and G-stage of kidney cancer. Employing the WGCNA algorithm package, we subsequently identified CLCN5 as the core gene within the brown module 27. Based on the survival analysis, only CLCN5 showed statistical significance among the nine family members in ccRCC. Notably, lower expression levels of CLCN5 were associated with poorer survival outcomes in ccRCC. Based on these results, we prioritized our focus on CLCN5 in the pathogenesis and progression due to the lack of previous reports in kidney cancer.

MicroRNAs/CLCN5 pathway might facilitate the evasion of apoptosis in chronic lymphocytic leukemia 8. CLCN5 had been reported to decrease the reabsorption of urinary proteins by renal tubular epithelial cells in patients with CLCN5 mutations 43, 44. In our study, CLCN5 expression was significantly reduced, and CLCN5 expression was negatively correlated with pathological grades and tumor stages in ccRCC. It was also an independent risk factor for overall survival in ccRCC patients. Subsequently, we conducted verification studies to assess the role of CLCN5 on kidney cancer cells. The transwell and cell proliferation experiments demonstrated that overexpression of CLCN5 significantly inhibited the growth and metastasis of ccRCC cells.

GSEA enrichment analysis uncovered a strong association between CLCN5 and fatty acid metabolism. The accumulation of lipids was considered a distinct feature of ccRCC and contributed to the progression and proliferation of ccRCC. Our previous research suggested that the overproduction of lipid droplets could be controlled by PLCL1/UCP1-mediated lipid browning process in ccRCC^45^. In this study, we found that CLCN5 overexpression could significantly reduce lipid storage in renal cancer cells. While previous reports had verified the role of UCP1, CPT1A, PPARA, and PTH in caner lipid metabolism 35-37, our study found that CLCN5 did not have an impact on these five genes' expression levels. Bioinformatics and cell experiments had demonstrated that EHHADH might be a unique downstream and mediated lipid degradation of CLCN5 in renal cancer cells. EHHADH, was a key gene in fatty acid metabolism of hepatocellular carcinoma 18, and oxidation of long-chain dicarboxylic acids (DCAs) 46. Diet with high fat induced obesity and promoted progression of lung cancer 47, lipid metabolism and metabolic reprogramming might contribute to cancer brain metastases 48 or cancer drug resistance 49, 50. Our studies found that CLCN5 played a role in influencing the progression of renal cancer through the promotion of fatty acid degradation in renal cancer cells. CLCN5 can significantly reduce lipid storage, and EHHADH may be a downstream of CLCN5 in renal cancer cells.

Although we have achieved certain research results of CLCN5/ EHHADH in ccRCC, further investigation is necessary due to the various limitations of the current study. Firstly, we have not validated the findings *in vivo* experiments, the specific mechanism of the influences of CLCN5 on EHHADH has not been carried out yet. Additionally, the potential link between chloride ion and lipid metabolism in renal cancer need to be further explored as CLCN5 is a chloride ion channel.

## Conclusions

This study elucidated the substantial importance of CLCN5 exhibited low expression levels and served as a biomarker of diagnostic and prognostic for ccRCC with bioinformatics analysis and clinical sample testing. Moreover, we have furnished preliminary evidence indicating that CLCN5 could inhibit the tumor proliferation, metastasis, migration, invasion, and promote lipid elimination in ccRCC. Furthermore, EHHADH acts as a downstream of CLCN5 in ccRCC. Therefore, the present research has unveiled a crucial regulatory mechanism for lipid metabolism of CLCN5/EHHADH signal in clear cell renal cell carcinoma.

## Supplementary Material

Supplementary figures and tables.

## Figures and Tables

**Table 1:** Relevant clinical data of ccRCC patients are all from the TCGA-KIRC database

	Parameter	Number	CLCN5 mRNA expression		P-value
			Low (n=259)	High (n=258)	
age	<= 60	258	140	118	0.065
	> 60	259	119	140	
gender	male	337	190	147	< 0.001*
	female	180	69	111	
T stage	T1+T2	330	144	186	< 0.001*
	T3 + T4	187	115	72	
N stage	N0 + NX	503	248	255	0.054
	N1	14	11	3	
M stage	M0 + MX	439	208	231	0.004*
	M1	78	51	27	
TNM stage	I + II	312	132	180	< 0.001*
	III + IV	205	127	78	
G stage	G1 + G2	238	101	137	0.001*
	G3 + G4	279	158	121	

The four-grid tables were made according to clinicopathological characteristics and The CLCN5 expression level. Statistical analyses were conducted via Pearson's χ2 test. *Indicates that the p value is statistically significant, p < 0.05.

**Table 2:** Relevant clinical data of ccRCC patients are all from the TCGA-KIRC database

	Univariate analysis			Multivariate analysis			Univariate analysis			Multivariate analysis		
	HR	95% CI	p	HR	95% CI	p	HR	95% CI	p	HR	95% CI	p
Overall survival (n= 517)												
CLCN5	0.474	0.347-0.648	< 0.001*	0.556	0.404-0.766	< 0.001*	0.431	0.296-0.627	< 0.001*	0.592	0.404-0.869	0.007
AGE	1.742	1.281-2.357	< 0.001*	1.774	1.299-2.420	< 0.001*	1.363	0.967-1.941	0.086			
GENDER	0.956	0.701-1.303	0.774				1.421	0.986-2.111	0.082			
T	3.038	2.243-4.114	< 0.001*				4.503	3.117-6.504	< 0.001*			
N	3.553	1.872-6.747	< 0.001*				5.915	22.969-11.781	< 0.001*	2.558	1.268-5.159	0.009
M	4.29	3.145-5.845	< 0.001*	2.399	1.657-3.472	< 0.001*	8.494	5.852-12.328	< 0.001*	4.012	2.630-6.120	< 0.001*
TNM	3.716	2.709-5.097	< 0.001*	1.904	1.290-2.810	< 0.001*	6.494	4.353-9.688	< 0.001*	2.801	1.748-4.490	< 0.001*
G STAGE	2.607	1.855-3.662	< 0.001*	1.615	1.125-2.317	0.009*	3.352	2.220-5.061	< 0.001*	2.263	1.478-3/465	< 0.001*

HR: Hazard ratio, estimated from Cox proportional hazard regression model CI: Confdence interval of the estimated HR Multivariate models were adjusted for T, N, M stage, G grade, age and gender * means the result is statistically significant

## Abbreviations

RCC: renal cancer carcinoma
ccRCC: clear cell renal cancer carcinoma
OS: overall survival
DFS: disease-free survival
TCGA-KIRC: The Cancer Genome Atlas kidney renal clear cell carcinoma
DEG: differentially expressed genes
WGCNA: weighted gene co-expression network analysis
KIRC: kidney renal clear cell carcinoma
TCGA: The Cancer Genome Atlas
CNA: copy-number alteration
GEO: gene expression omnibus
ROC: receiver operator characteristic
qRT-PCR: reverse transcription-quantitative PCR
IHC: immunohistochemistry
WB: western blot
CCK-8: cell counting kit-8
GSEA: gene set enrichment analysis
GO: gene ontology
CIC: chloride ion channels
KEGG: Kyoto Encyclopedia of Genes and Genomes
PBST: phosphate buffered saline tween
FDR: false discovery rate
SEM: standard error of measurement
FAO: fatty acid β-oxidation
DCAs: dicarboxylic acids
