# Supplementary material for: CLCN5 inhibits tumorigenesis and fatty acid accumulation in clear cell renal cell carcinoma by regulating Enoyl CoA hydratase and 3-Hydroxyacyl CoA dehydrogenase
Source: Int J Med Sci. 2025 Jul 19;22(13):3354–70. doi: 10.7150/ijms.105969 (PMC12320790; doi:10.7150/ijms.105969)
Supplement: Supplementary file 1 — Supplementary figures and tables. [file ijmsv22p3354s1.pdf]

# CLCN5 inhibits tumorigenesis and fatty acid accumulation in clear cell renal cell carcinoma by regulating Enoyl CoA hydratase and 3-Hydroxyacyl CoA dehydrogenase

Tiexi Yu[a,b,c,*], Weiquan Li[a,b,c,*], Xiangui Meng [a,b,c,*], Wei Yang[a,b,c], Hailong Ruan[a,b,c,#], Wen Xiao [a,b,c,#], Xiaoping Zhang [a,b,c,#]

[*] Tiexi Yu, Weiquan Li, and Xiangui Meng contributed equally to this work

[#] Correspondence:

Hailong Ruan hlruan2018@hust.edu.cn

Wen Xiao wxuro20@hust.edu.cn

Xiaoping Zhang xzhang@hust.edu.cn

[a] Department of Urology, Union Hospital, Tongji Medical College, Huazhong University of Science and Technology, Wuhan, 430022, China.

[b] Shenzhen Huazhong University of Science and Technology Research Institute, Shenzhen 518000, China.

[c] Institute of Urology, Tongji Medical College, Huazhong University of Science and Technology, Wuhan 430022, China.

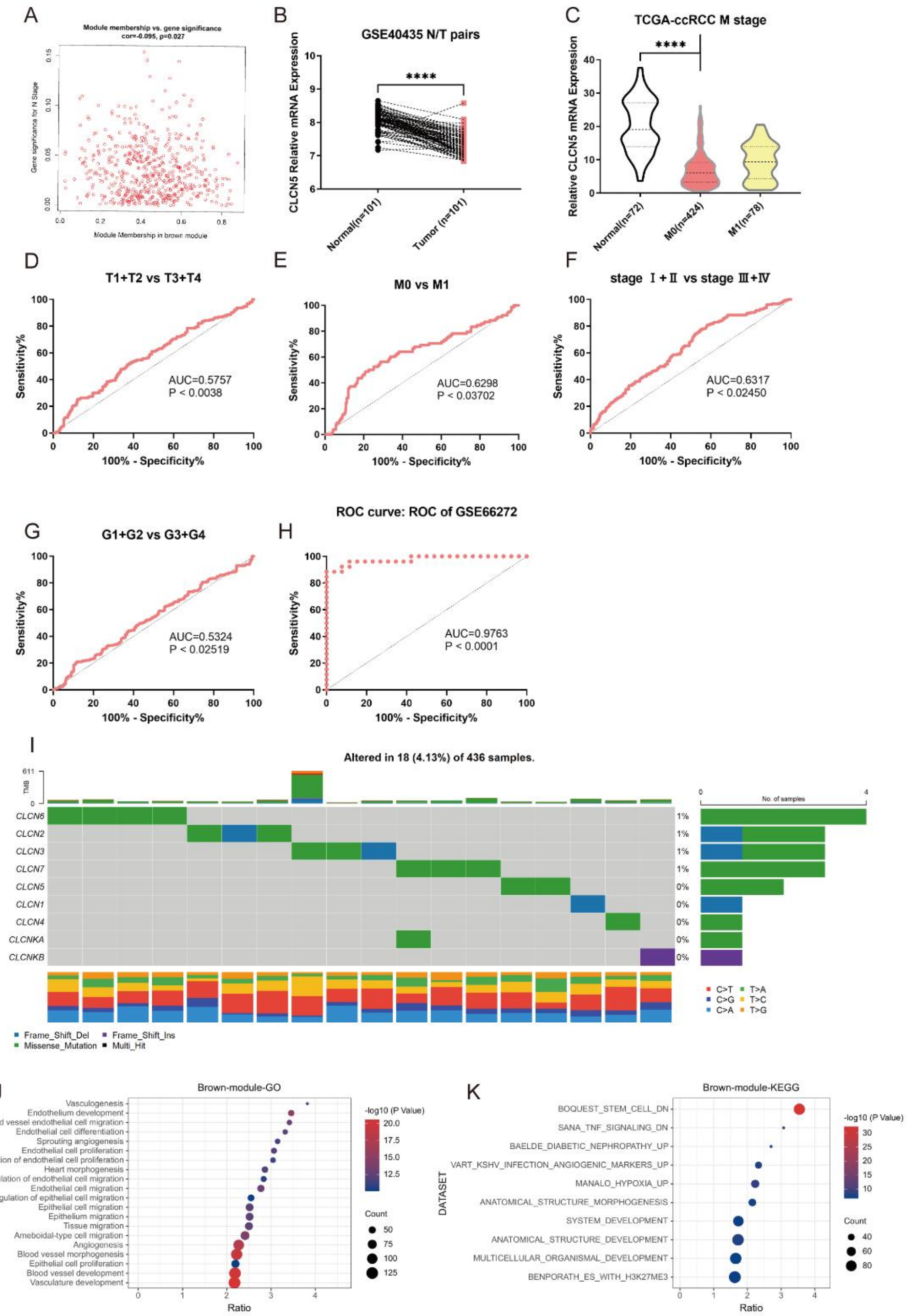


**Supplement FigureS1:** A: The relationship between the brown module and the N stage. B: CLCN5 expression level between normal and tumor in GSE40435 dataset. C: CLCN5 expression level both. M stage in GSE40435 dataset. D-F: The ROC curve between clinicopathological subgroups in the TCGA-KIRC dataset. H: ROC curves of GSE66272 dataset. I: The copilot of CIC family members.  J, F: The GO and KEGG of the brown module identified by WGCNA.

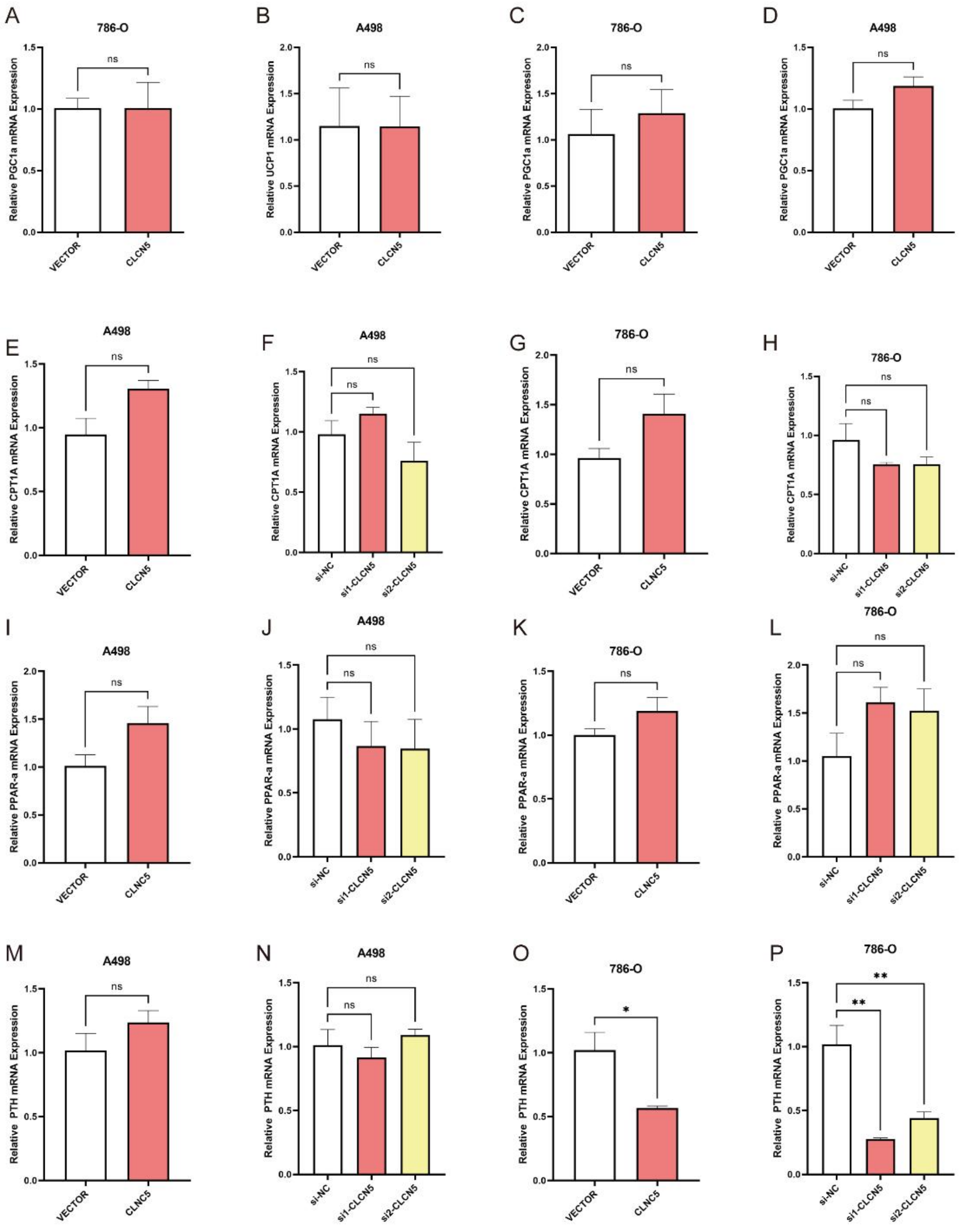


**Supplement FigureS2:** A-P: the mRNA level of classical genes associated with lipid metabolism (UCP1, CPT1A, PPARA, PTH, and PGC1A) in up-regulated and down-regulated CLCN5 in A498 and 786-O cell line.

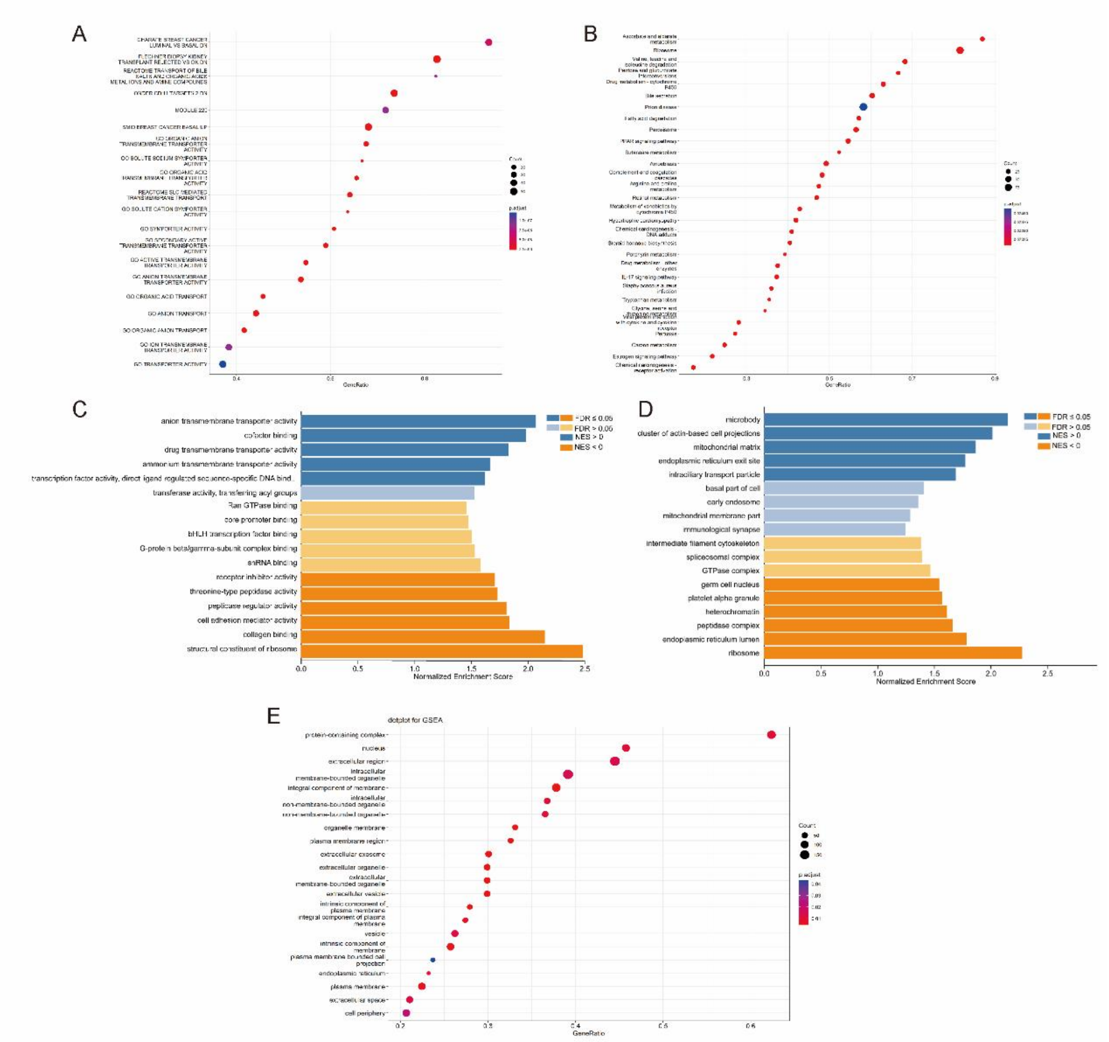


**Supplement FigureS3:** A, B, E: The GO, KEGG, and GSEA of CLCN5 illustrated by R. C, D: The MF (molecular function) and CC (Cell Component) of CLCN5.

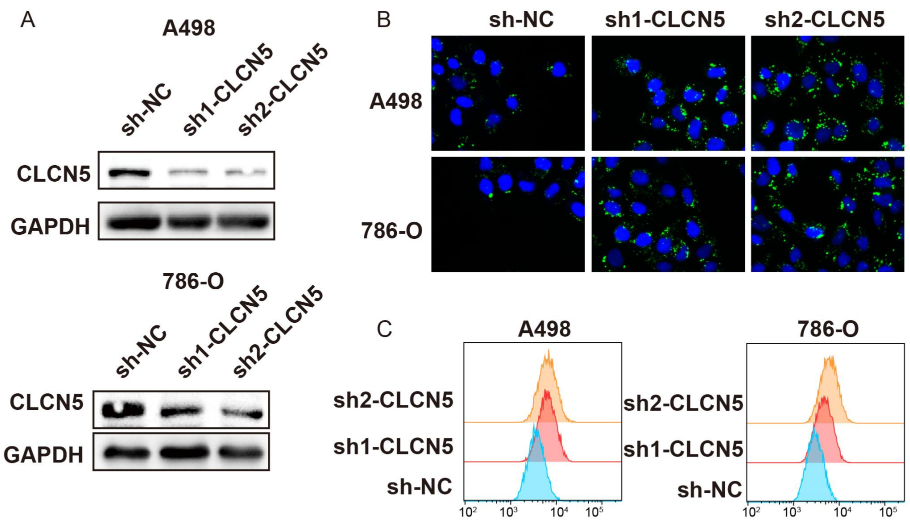


**Supplement FigureS4:** A, Stable knockdown CLCN5 was constructed in renal cancer cell lines; B and C: Fluorescence microscopy analysis and neutral lipid levels were showed the function of stable knockdown CLCN5 in renal cancer cells.
